# Cryoinjuries in Cryopreserved Semen of *Ichthyoelephas longirostris* with Ethylene Glycol

**DOI:** 10.3390/ani15162338

**Published:** 2025-08-10

**Authors:** Jaider Alonso Martínez-Suarez, José Alonso Espinosa-Araujo, Víctor Atencio-García

**Affiliations:** 1 Master’s Program in Veterinary Sciences of the Tropics, Faculty of Veterinary Medicine and Animal Science, University of Córdoba, Montería 230001, Colombia; jmartinezsuarez@correo.unicordoba.edu.co; 2 CINPIC, Fish Culture Research Institute, Department of Aquaculture Sciences, Faculty of Veterinary Medicine and Animal Science, University of Córdoba, Montería 230001, Colombia; jaespinosaaraujo@correo.unicordoba.edu.co

**Keywords:** DNA, membrane, mitochondria, prochilodontid, spermatozoa

## Abstract

*Ichthyoelephas longirostris*, commonly known as pataló, is an endemic species from Colombia, categorized as endangered due to habitat degradation and overfishing. In this context, semen cryopreservation emerges as an effective strategy for its conservation. This study evaluated the damage caused by semen cryopreservation using a cryosolution composed of ethylene glycol (EG) at three inclusion levels (6%, 8%, and 10% *v*/*v*), skimmed milk powder (3% *w*/*v*), and glucose (6% *w*/*v*), all prepared in distilled water. A randomized experimental design was employed, with three treatments and a control (fresh semen), using pooled semen from different males (*n* = 12). Each sample was analyzed in triplicate. Sperm parameters were evaluated through computer-assisted analysis (CASA), and damage to the plasma membrane, mitochondria, and DNA was assessed via flow cytometry. Fresh semen showed low levels of membrane damage (9.6 ± 6.9%), mitochondrial damage (29.1 ± 16.8%), and DNA fragmentation (0.24 ± 0.13%). In thawed semen, the highest membrane damage was observed with 10% EG (79.3 ± 13.0%), while the highest DNA fragmentation occurred with 6% EG (22.5 ± 21.9%). The cryosolution composed of 8% EG showed the best results, with lower levels of damage in the evaluated structures. These findings suggest that the combination of 8% EG, 3% skimmed milk powder, and 6% glucose is an effective formulation to reduce cellular damage during *I. longirostris* semen cryopreservation, representing a useful tool for conservation programs of this species.

## Simple Summary

Anthropogenic activity, particularly overfishing, has led to a marked decline in commercially important fish populations in the Cauca River, particularly affecting the Pataló (*Ichthyoelephas longirostris*). The objective of this study was to evaluate membrane, mitochondrial, and DNA damage in Pataló semen cryopreserved with ethylene glycol at three different concentrations. Sperm motility parameters were analyzed using a computer-assisted analysis system; cryodamage was measured using flow cytometry. In thawed semen, the highest total motility values were recorded with 6% and 8% ethylene glycol. However, the highest percentages of mitochondrial and membrane damage were observed when 10% ethylene glycol was added. DNA fragmentation was highest with 6% ethylene glycol. The results suggest that 8% ethylene glycol is the most suitable cryosolution for the cryopreservation of Pataló semen, as it offers a better balance between sperm motility and cell protection. This study contributes to the conservation of hydrobiological resources in Colombia and, therefore, to food security and the economic livelihood of fishing communities.

## 1. Introduction

*Ichthyoelephas longirostris*, commonly known as pataló, is an endemic species of the Magdalena-Cauca and Ranchería river basins [1,2]. It mainly inhabits the tributaries of the large rivers within the Magdalena basin, preferring clear, fast-flowing waters, and is less common in floodplain lakes [3]. It is a short-distance migratory species (<100 km) during the dry season, moving downstream to larger rivers when the waters become less turbid [4]. Its populations are currently threatened by activities such as mining, deforestation, organic and inorganic pollution, and high fishing pressure, especially in the upper Cauca River basin, where its meat is highly prized. In some cases, unsustainable or illegal extraction methods are used [3].

In light of this situation, urgent management and conservation actions are required, both in situ and ex situ. In the latter approach, the implementation of biotechnological tools is essential for the conservation of the species. Fish semen cryopreservation represents an effective strategy for preserving genetic material, especially in threatened or endangered species [5,6]. Furthermore, this technique facilitates the transfer of semen between fish farming stations, helps resolve issues related to reproductive asynchrony in aquaculture, and provides valuable material for genetic research and conservation efforts [7,8].

Cryopreservation protocols are a set of procedures with defined operational parameters aimed at preserving viable cells over the long term [7]. Although this technique ensures sperm survival, it can induce structural and functional damage during the process, affecting the nucleus, midpiece, flagellum, plasma membrane, and cytoplasm. These damages compromise DNA integrity, induce mitochondrial dysfunction, affect cellular metabolism, reduce sperm motility and velocity, and lead to the loss of proteins essential for fertilization [5,9,10,11]. Such alterations are associated with thermal, osmotic, and oxidative stress experienced by the cell during dilution in the cryosolution, as well as during freezing and thawing [5,11,12,13].

For a fish semen cryopreservation protocol to be successful, it is essential to optimize each of the critical factors in the process, including semen collection, the composition of the extender, the concentration of the cryoprotectant, and the freezing and thawing curves [14,15,16,17,18]. Ethylene glycol (EG) is a cryoprotectant with membrane permeability that has proven effective in freshwater fish species [19,20], including members of the Prochilodontidae family [21]. In this study, the damage to cryopreserved semen of *I. longirostris* was evaluated using a cryosolution composed of EG at three inclusion rates (6%, 8%, and 10%), 3% skim milk powder (SMP), and 6% glucose, as an in vitro ex situ conservation strategy for this endangered species.

## 2. Materials and Methods

### 2.1. Study Area Location, Animals, and Semen Collection

The research was conducted at the Fish Research Institute of the University of Córdoba (CINPIC), located in the municipality of Montería, department of Córdoba, with geographical coordinates: 8°47.5′ North Latitude and 75°51′ West Longitude.

Twelve specimens of *I. longirostris* were used, with an average weight of 0.7 ± 0.1 kg and a total length (TL) of 40.3 ± 1.3 cm. They were captured in the Espíritu Santo River (Briceño, Antioquia, Colombia), adapted, and kept in captivity for six months in earthen ponds at a density of 1 fish/m^2^ at CINPIC. Males in the spermiation phase were selected, identified by the presence of seminal fluid in the urogenital papilla after slight abdominal pressure in a cranio-caudal direction. The selected specimens were transferred to circular tanks of 6 m^3^, where they remained for 48 h to acclimate to experimental conditions and reduce stress caused by handling, environmental changes, and the administration of a hormonal inducer to stimulate an increase in seminal volume. The dose of 4.8 mg/kg of carp pituitary extract (Argent, Ruston, LA, USA) was selected based on previous protocols applied to other prochilodontids [21].

Spermiation was confirmed 6 h after hormone administration by the continuous release of semen under gentle abdominal pressure. Semen was collected at this time. Before semen extraction, the fish were sedated with 2-phenoxyethanol (300 ppm, Sigma Chemical, St. Louis, MI, USA). Immediately after loss of swimming equilibrium, the fish were removed, and the ventral area was dried. Gentle pressure was then applied to expel any urine. The quality of the semen collected after hormonal treatment (fresh semen) was used to compare with the quality of thawed semen, following the protocols described for fish semen evaluation [22,23].

The study was conducted in accordance with the Code of Conduct for the Use of Animals in Teaching and Research established by the Central Bioethics Committee (Technical Knowledge Panel of Veterinary Medicine and Animal Science) of the University of Córdoba (Colombia) (11 August 2023, CW246358).

### 2.2. Sperm Kinematics

Total motility (TM), progressivity, and sperm velocities. A volume of 0.25 μL of semen was placed in the central area of a Makler chamber (Sefi Medical Instruments, Haifa, Israel), and immediately diluted in situ with 75 μL of distilled water (1:300 dilution). The sample was then analyzed using a computer-assisted sperm analysis system (CASA) (Microptic SL, SCA^®^ VET 6.5, Barcelona, Spain) and a phase contrast optical microscope (Nikon, E50i, Tokyo, Japan) with a 10× objective. The CASA system analyzed three fields per sample, and in each field, a minimum of 500 sperm were analyzed. Sperm motility was classified as rapid (type a) when velocities were greater than 100 μm/s, medium (type b) for velocities between 50 and 100 μm/s, and slow (type c) for velocities below 50 μm/s [22,24]. Curvilinear velocity (VCL) was defined as the total distance traveled over time (μm/s) along the actual sperm trajectory between two points, while straight-line velocity (VSL) referred to the linear path from the first to the last point [22]. Velocity-related indices such as linearity (LIN), straightness (STR), and wobble (WOB) were also estimated, along with head displacement parameters like amplitude of lateral head displacement (ALH) and beat cross frequency (BCF) [22].

The CASA system was previously adjusted and validated for Ichthyoelephas longirostris. Key parameters such as frame rate (50 Hz), number of frames [25], minimum sperm size (10 µm), and velocity thresholds were optimized using fresh semen samples in preliminary trials. The movement patterns were visually confirmed under phase contrast microscopy to ensure accurate tracking. The parameters (VCL, VSL, VAP, LIN, STR, etc.) were analyzed only when the sperm trajectories were clearly distinguishable from static particles or debris, ensuring data reliability.

### 2.3. Treatments, Freezing, and Thawing of Semen

The cryosolution was composed of ethylene glycol (EG) at three inclusion levels (6%, 8%, and 10% *v*/*v*), skimmed milk powder (3% *w*/*v*), and glucose (6% *w*/*v*), all prepared in distilled water. Fresh semen was used as the control treatment to compare the quality of the thawed semen. It is important to note that the analysis of fresh semen was performed approximately 8 h after collection. This time lapse could affect certain sperm parameters and is acknowledged as a limitation of the study. However, the samples were kept at room temperature, which is considered more suitable for semen handling in this species.

The semen was diluted at a 1:4 ratio (semen: diluent) [25] and packed in 0.25 mL straws. Prior to freezing (pre-frozen semen), the total motility percentage was evaluated as previously described. The straws (Minitüb, Tiefenbach, Germany) were sealed with polyvinyl alcohol (PVA) (Alquera, Madrid, Spain) and placed in a dry shipper (MVE, SC 4/2v, Ball Ground, GA, USA) for 30 min to freeze them in nitrogen vapors. The freezing curve in the dry shipper was described as follows: from 28 to −20 °C at a rate of 29.9 °C/min, from −20 to −100 °C at a rate of 27.3 °C/min, and from −100 to −196 °C at a rate of 5.5 °C/min [16]. Afterwards, the straws were transferred to a cryogenic storage tank (MVE XC 34/18, Ball Ground, GA, USA), fully submerged in liquid nitrogen. After six months, post-thaw semen quality was evaluated. Thawing was performed in a serological bath (Mermmet GmbH & Co. KG, Schwabach, Germany) at 35 °C for 60 s [26].

### 2.4. Cryodamage in Mitochondria, Plasma Membrane, and DNA

Mitochondrial function damage (Mit-d) and plasma membrane damage (Mem-d) were evaluated in fresh and thawed semen using aliquots of 1 and 10 µL, respectively. Sample staining was performed by adding 1 mL of a solution containing 3,3′-dihexyloxacarbocyanine iodide (DiOC6(3)) (70 nM) and propidium iodide (PI) (2 µg/mL) (Sigma, St. Louis, MO, USA), incubating in the dark for 20 min, and then analyzing the samples by flow cytometry. DiOC6(3) staining allowed the identification of cells with a high uptake of DiOC6(3) (viable cells) and low uptake (mitochondrial damage without sperm membrane damage). PI staining indicated loss of integrity in the plasma membrane [27].

DNA fragmentation (DNA-f) was analyzed in fresh and thawed sperm using aliquots of 10 and 100 µL, respectively. Samples were fixed with 3 mL of 70% ethanol (Sigma, St. Louis, MO, USA), incubated for 12 h at 20 °C, washed with calcium-free, ultrafiltered BD PBS, vortexed, and centrifuged at 16,163× *g* for 10 min at 4 °C. The supernatant was discarded, and the pellet was resuspended in 300 µL of propidium iodide (PI) + RNase A (20 U) per million cells. The samples were vortexed again and incubated in the dark for 20 min at room temperature before flow cytometry analysis [27].

For DiOC6(3) and PI tests, the detector voltage settings were specifically optimized for this assay using unlabeled cells, with the acquisition threshold defined as a function of the cell suspension medium (PBS without seminal fluid), to minimize background noise. Cell population identification and gating were performed using a forward scatter (FSC) versus side scatter (SSC) plot, which enabled the identification of the sperm population based on their size and internal complexity. In this step, events corresponding to cellular debris, detritus, or non-sperm material were excluded, as low-intensity signals in both parameters characterized these types of events. This strategy ensured that subsequent analysis was performed exclusively on the target sperm population. Aggregates were excluded by discriminating doublets with FSC-H vs. FSC-A graphics.

Fluorescence compensation was required due to spectral overlap between the DiOC6(3) (530/30 nm) and PI (610/30 nm) channels. Single staining was used for each fluorochrome, with compensation matrices automatically generated using FlowJo v10.9 software. Manual adjustments were made where necessary to align fluorescence medians with those of negative controls (unstained cells). FMO controls were not employed, as single-stain controls were used, and a two-fluorochrome experimental design with minimal additional overlap was used.

For DNA fragmentation assessment, the detection threshold was established based on the fluorescence distribution of the negative control (unstained cells), classifying as fragmented those cells whose fluorescence intensity exceeded the upper limit of this distribution. Doublet discrimination was performed in two steps: initially using fluorescence signal amplitude and width parameters (PI-W vs. PI-H at 610/30 nm), and then using FSC-H vs. FSC-A. In addition, chicken erythrocytes and sheep T cells were incorporated as linearity and coefficient of variation controls, included in the BD DNA QC kit (BD Biosciences, Franklin Lakes, NJ, USA).

Flow cytometry analysis was performed on an LSRFortessa™ | High-Parameter Flow Cytometer (BD Biosciences) with a 488 nm excitation laser and detection filters appropriate for each fluorophore, as indicated above. At least 10,000 events were collected per sample. Data acquisition and analysis were performed using FACSDiva (System: Windows 7 version 6.1) and FlowJo (v10.9) software, respectively. Each treatment was evaluated in three biological replicates, and each sample was processed in duplicate as a technical replicate to ensure the reproducibility of the analysis.

**Figure 1:**
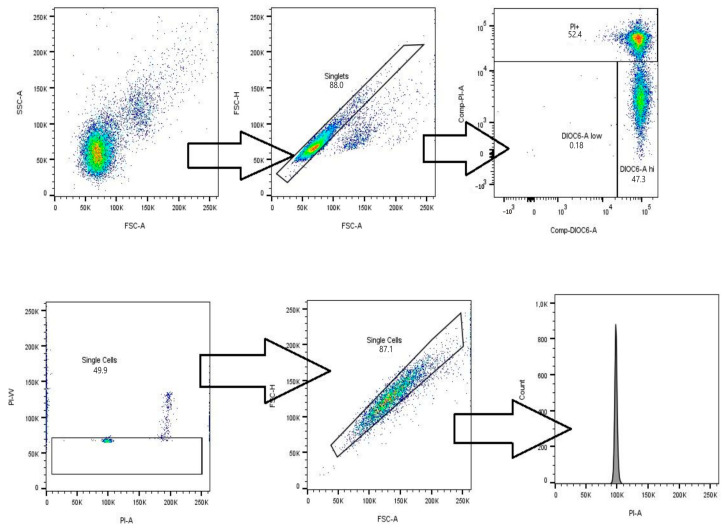
Flow cytometry analysis for the evaluation of cell viability, mitochondrial integrity, and DNA fragmentation.

Figure 1 illustrates the key events of the analysis: initial cell population selection (FSC-A vs. SSC-A), singlet discrimination (FSC-A vs. FSC-H), selection of viable and mitochondrially capable cells (DiOC6 vs. PI), and quantification of cells with fragmented DNA using PI fluorescence intensity. The threshold for classifying cells with fragmented DNA was established based on negative controls and the shift in the sub-G1 peak in the histogram.

### 2.5. Statistical Analysis

Semen was collected from several mature males, pooled to reduce individual variability, and then evenly distributed among the experimental treatments, ensuring biological representation across groups. Each treatment included three biological replicates, and each replicate was analyzed in triplicate as technical replicates. A completely randomized design was used for treatment assignment and sample processing to minimize potential bias in the results. All data were subjected to normality tests (Shapiro–Wilk test) and homogeneity of variance tests (Levene’s test). Once these assumptions were met, an analysis of variance (ANOVA) was performed, and when significant differences were found, Tukey’s multiple range test was applied. In all cases, *p* < 0.05 was considered the level of significance. Additionally, a Pearson correlation test was conducted to explore linear relationships between semen quality variables and cryodamage. The results were visualized in a correlation matrix (heatmap). All analyses were performed using the R statistical software, R Studio version. 4.2.3.

## 3. Results

### 3.1. Quality of Fresh and Thawed Semen

Table 1 shows the sperm kinematic values in fresh and thawed semen of *I. longirostris*. As expected, fresh semen recorded the best sperm kinematic values, being statistically different from those obtained in thawed semen (*p* < 0.05). In thawed semen, total motility (TM) was higher when ethylene glycol (EG) was included at 6% and 8%, with no statistical difference observed between these two values (*p* > 0.05). Furthermore, at these inclusion levels (6% and 8%), thawed semen showed the lowest percentages of immotile spermatozoa (*p* > 0.05), whereas when EG was included at 10%, the lowest percentage of slow-moving spermatozoa (40.0 ± 2.8%) was obtained, being statistically different from the other treatments (*p* < 0.05).

In thawed semen, no statistically significant differences were observed among the different EG inclusion levels (6%, 8%, and 10%) for parameters such as velocities (VCL, VSL, and VAP), velocity indices (LIN, STR, and WOB), and head angularity and oscillation parameters (ALH and BCF).

### 3.2. Membrane Damage, Mitochondrial Damage, and DNA Fragmentation

Table 2 shows cryodamage to the membrane (Mem-d), mitochondria (Mit-d), and DNA fragmentation (DNA-f) in the fresh and thawed semen of *Ichthyoelephas longirostris*. Fresh semen showed the lowest Mem-d values (9.6 ± 6.9%) (*p* < 0.05). In thawed semen, the highest percentages of Mem-d (79.3 ± 13.0%) and Mit-d (81.0 ± 18.8%) were recorded when EG was included at 10%, with statistical differences observed compared to the other treatments (*p* < 0.05). It is important to highlight that fresh semen showed Mit-d (29.1 ± 16.8%, *p* < 0.05) higher than that recorded in thawed semen when EG8% (4.0 ± 4.9%) and EG10% (1.2 ± 0.5%) were used. The highest DNA-f values were observed with EG6% (22.5 ± 21.9%), with significant statistical differences compared to the other treatments (*p* < 0.05); however, no statistical difference (*p* > 0.05) was found between DNA-f in fresh semen (0.24 ± 0.13%) and thawed semen with EG8% (0.30 ± 0.13%) and EG10% (0.48 ± 0.39%).

**Figure 2:**
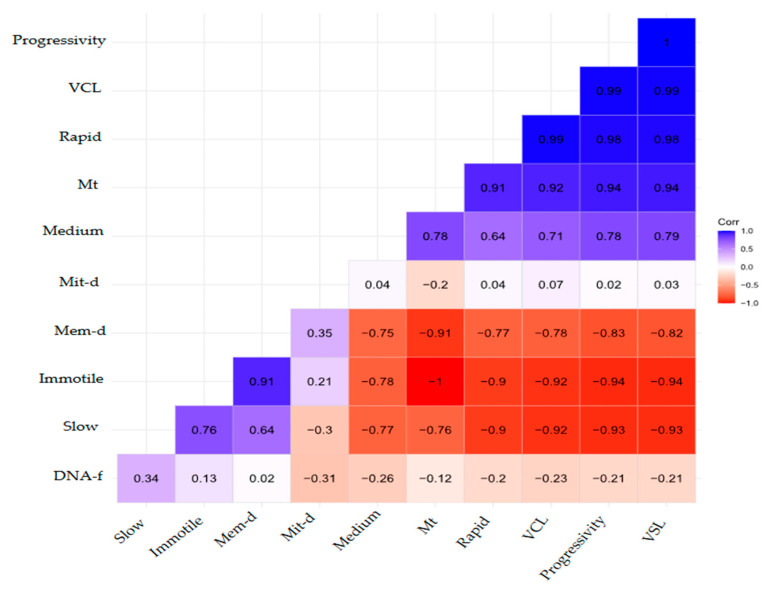
Correlation between sperm quality parameters and cryodamage in *I. longirostris* semen. VCL: curvilinear velocity, Mt: total motility, Mit-d: mitochondrial damage, Mem-d: membrane damage, DNA-f: DNA fragmentation, VSL: straight-line velocity.

Figure 2 shows the correlation between sperm quality parameters and cryodamages in *I. longirostris* semen. A strong negative correlation was observed between total motility and damage to the cell membrane.

## 4. Discussion

Sperm kinematics comprise a set of parameters that allow for the inference of the fertilizing capacity of the spermatozoon. Among these, sperm motility is defined as the ability of the sperm to move and locate the oocyte, which is essential for successful fertilization [28]. This variable, which reflects movement in any direction, is considered one of the main indicators for evaluating semen quality and has been recognized as the best bioindicator of sperm quality in fish [29,30].

In the present study, the total motility of fresh semen from *I. longirostris* exceeded 90%, indicating high semen quality. According to the literature, total motility above 80% is a reliable criterion for classifying an ejaculate as of good quality [31]. These results suggest that the males adapted adequately to the captivity conditions. Similar values have been reported in other prochilodontids such as *Prochilodus magdalenae* [21,24], *Prochilodus brevis* [32,33], and *Prochilodus lineatus* [34,35], as well as in Characiformes of the genus *Brycon*, including *Brycon sinuensis* [36] and *Brycon orbignyanus* [37].

Other parameters considered in sperm kinetics include curvilinear velocity (VCL), straight-line velocity (VSL), and average path velocity (VAP), as well as the coefficients of straightness, wobble, amplitude of lateral head displacement, and beat-cross frequency. In general, the values obtained for fresh *I. longirostris* semen fell within the ranges previously reported for freshwater fish from the Neotropics.

At the evaluated inclusion percentages (EG6%, EG8%, and EG10%), significant differences were found only in total motility and in the percentage of slow and immotile spermatozoa. *I. longirostris* semen cryopreserved with 10% EG exhibited the lowest total motility, with statistical differences compared to those cryopreserved with 6% and 8% EG. Likewise, the highest proportion of immotile spermatozoa was recorded in the 10% EG treatment. These results suggest that both the type of cryoprotectant and its concentration directly influence post-thaw sperm motility [38], indicating that a 10% concentration of EG may be toxic to *I. longirostris* spermatozoa.

VCL, VSL, and VAP values presented in Table 1 showed no statistical differences between treatments and were within the ranges reported for other prochilodontids such as *Prochilodus brevis* [32,39]. Nevertheless, the cryopreservation and thawing process caused a general reduction in total motility, ranging from 36.5% (EG8%) to 58.8% (EG10%). Similarly, decreases in sperm velocities, overall progressivity, and an increase in the percentage of immobile spermatozoa were observed in the thawed semen.

Regarding plasma membrane damage, the values recorded for both fresh and thawed semen were similar to those reported in other characiforms such as *Prochilodus magdalenae* [40], *P. lineatus* [41], and *P. brevis* [39]. During the freezing and thawing process, cell membranes serve as the first line of defense against abrupt changes in temperature and osmolarity, making them particularly vulnerable [7]. The loss of membrane lipid fluidity is one of the factors contributing to structural damage [42]. Furthermore, membrane integrity can be compromised by sperm dehydration, intracellular ice crystal formation, physical membrane rupture, as well as osmotic and oxidative stress [40,43,44].

The highest mitochondrial damage was observed in semen cryopreserved with 10% EG, showing significant differences compared to the other treatments. This indicates that a higher concentration of the cryoprotectant may compromise mitochondrial functionality, likely due to its toxic effect. It is worth noting that fresh semen also showed significant differences compared to the treatments with 6% and 8% EG. This finding could be attributed to the time elapsed before analysis, since due to logistical difficulties, the evaluation of fresh semen was performed eight hours after collection. This delay may have induced oxidative processes, especially at the mitochondrial level, considering that these organelles are major sources of reactive oxygen species (ROS) [45]. In contrast, the thawed samples were evaluated immediately after thawing, which likely minimized exposure to oxidative stress. This highlights the importance of defining a maximum time between the collection and analysis of fresh semen to avoid underestimating its mitochondrial quality.

Regarding DNA integrity, the observed damage may be related to ice crystal formation, osmotic stress, or oxidative stress, which can induce DNA breakage through mechanical pressure or the activation of apoptotic mechanisms during the freezing and thawing process [46]. In this study, DNA fragmentation was evaluated, and it was found that the treatment with 6% EG showed a higher level of damage compared to the 8% and 10% EG levels. The latter did not show significant differences compared to fresh semen, suggesting they provided better protection of the genetic material during the semen freezing and thawing process.

In general, the greatest cryodamage to membranes and mitochondria was observed in the treatment with 10% EG, which was also reflected in the reduced total motility values. This toxicity could be associated with an excess of intracellular solutes or the triggering of programmed cell death (apoptosis). It has been documented that structural changes in the plasma membrane during cryopreservation are closely linked to the type and concentration of the cryoprotectant, as well as the composition of the diluent, factors that can activate apoptotic pathways [27]. However, in terms of DNA fragmentation, the values obtained with 10% EG were comparable to those with 8% EG, suggesting that despite greater mitochondrial and membrane damage, the cell nucleus maintained relative integrity.

## 5. Conclusions

The results of the present study indicate that the cryoprotective solution with 10% EG caused greater damage to the mitochondria and plasma membrane of *I. longirostris* spermatozoa, while DNA fragmentation was comparable to that observed with 8% EG. In contrast, the treatment with 6% EG caused a higher level of DNA fragmentation. Therefore, it is suggested that 8% ethylene glycol represents the most adequate concentration among those evaluated, as it better preserves sperm kinetics and offers greater protection at the level of the plasma membrane, mitochondria, and DNA integrity during the cryopreservation process.

## Figures and Tables

**Table 1:** Quality of fresh and thawed semen of *Ichthyoelephas longirostris* cryopreserved with ethylene glycol (EG) at three inclusion percentages (6%, 8%, and 10%).

Parameters	Fresh Semen	Thawed Semen		
		EG6%	EG8%	EG10%
Total motility (%)	93.3 ± 6.8 ^a^	55.0 ± 6.7 ^b^	59.3 ± 1.9 ^b^	45.9 ± 3.9 ^c^
Progressivity (%)	64.4 ± 17.2 ^a^	0.7 ± 0.1 ^b^	2.6 ± 0.8 ^b^	1.3 ± 0.6 ^b^
Rapid (%)	54.0 ± 21.2 ^a^	0.1 ± 0.0 ^b^	1.7 ± 0.6 ^b^	0.6 ± 0.2 ^b^
Medium (%)	24.1 ± 11.0 ^a^	1.4 ± 0.2 ^b^	4.9 ± 1.4 ^b^	5.3 ± 1.5 ^b^
Slow (%)	15.2 ± 8.9 ^c^	53.3 ± 6.5 ^a^	52.6 ± 0.5 ^a^	40.0 ± 2.8 ^b^
Immotile (%)	6.2 ± 6.6 ^c^	45.0 ± 6.7 ^ab^	40.1 ± 1.5 ^b^	54.1 ± 3.9 ^a^
VCL (µm/s)	103.9 ± 22.4 ^a^	19.6 ± 1.4 ^c^	26.4 ± 2.8 ^bc^	25.7 ± 1.0 ^bc^
VSL (µm/s)	61.3 ± 15.4 ^a^	2.1 ± 0.1 ^b^	3.9 ± 0.8 ^b^	3.7 ± 0.4 ^b^
VAP (µm/s)	89.2 ± 20.7 ^a^	6.1 ± 0.1 ^b^	9.1 ± 1.2 ^b^	9.2 ± 0.3 ^b^
LIN	54.0 ± 7.2 ^a^	9.3 ± 1.3 ^b^	10.6 ± 0.5 ^b^	10.7 ± 1.2 ^b^
STR	63.2 ± 5.5 ^a^	36.3 ± 1.9 ^b^	38.0 ± 0.7 ^b^	33.9 ± 2.5 ^b^
WOB	81.2 ± 5.0 ^a^	29.8 ± 2.2 ^b^	29.5 ± 0.1 ^b^	30.5 ± 1.0 ^b^
ALH	1.6 ± 0.3 ^a^	0.8 ± 0.0 ^b^	1.0 ± 0.1 ^b^	0.9 ± 0.1 ^b^
BCF	11.2 ± 2.7 ^a^	0.7 ± 0.0 ^b^	1.0 ± 0.1 ^b^	1.1 ± 0.1 ^b^

VCL, curvilinear velocity, VSL, straight-line velocity, VAP, average path velocity, LIN, linearity, STR, straightness, WOB, wobble coefficient, ALH, amplitude of lateral head displacement, BCF, beat cross frequency. Different lowercase letters indicate statistically significant differences

**Table 2:** Membrane Damage (Mem-d), Mitochondrial Damage (Mit-d), and DNA Fragmentation (DNA-f) in Fresh and Thawed Semen of *Ichthyoelephas longirostris*.

Damages	Fresh Semen	EG6%	EG8%	EG10%
Mem-d (%)	9.6 ± 6.9 ^a^	59.6 ± 6.8 ^b^	50.4 ± 6.0 ^b^	79.3 ± 13.0 ^c^
Mit-d (%)	29.1 ± 16.8 ^b^	4.0 ± 4.9 ^a^	1.2 ± 0.5 ^a^	81.0 ± 18.8 ^c^
DNA-f (%)	0.24 ± 0.13 ^a^	22.5 ± 21.9 ^b^	0.30 ± 0.13 ^a^	0.48 ± 0.39 ^a^

Different lowercase letters indicate statistically significant differences.

## Data Availability

All data provided in this manuscript were appropriately cited in the tables, figures, and references sections.
